# Determinants of Healthcare Utilisation and Out-of-Pocket Payments in the Context of Free Public Primary Healthcare in Zambia

**DOI:** 10.15171/ijhpm.2016.65

**Published:** 2016-06-01

**Authors:** Felix Masiye, Oliver Kaonga

**Affiliations:** Department of Economics, University of Zambia, Lusaka, Zambia.

**Keywords:** Access to Healthcare, Health, Expenditure, Removal of User Fees, Zambia

## Abstract

**Background:** Access to appropriate and affordable healthcare is needed to achieve better health outcomes in Africa. However, access to healthcare remains low, especially among the poor. In Zambia, poor access exists despite the policy by the government to remove user fees in all primary healthcare facilities in the public sector. The paper has two main objectives: (i) to examine the factors associated with healthcare choices among sick people, and (ii) to assess the determinants of the magnitude of out-of-pocket (OOP) payments related to a visit to a health provider.

**Methods:** This paper employs a multilevel multinomial logistic regression to model the determinants of an individual’s choice of healthcare options following an illness. Further, the study analyses the drivers of the magnitude of OOP expenditure related to a visit to a health provider using a two-part generalised linear model. The analysis is based on a nationally representative healthcare utilisation and expenditure survey that was conducted in 2014.

**Results:** Household per capita consumption expenditure is significantly associated with increased odds of seeking formal care (odds ratio [OR] = 1.12, *P* = .000). Living in a household in which the head has a higher level of education is associated with increased odds of seeking formal healthcare (OR = 1.54, *P* = .000) and (OR = 1.55, *P* = .01), for secondary and tertiary education, respectively. Rural residence is associated with reduced odds of seeking formal care (OR = 0.706, *P* = .002). The magnitude of OOP expenditure during a visit is significantly dependent on household economic well-being, distance from a health facility, among other factors. A 10% increase in per capita consumption expenditure was associated with a 0.2% increase in OOP health expenditure while every kilometre travelled was associated with a K0.51 increase in OOP health expenditure.

**Conclusion:** Despite the removal of user fees on public primary healthcare in Zambia, access to healthcare is highly dependent on an individual’s socio-economic status, illness type and region of residence. These findings also suggest that the benefits of free public healthcare may not reach the poorest proportionately, which raise implications for increasing access in Zambia and other countries in sub-Saharan Africa.

## Background

Expanded access to healthcare is now well-recognised as critical to achieving better health outcomes and economic development in sub-Saharan Africa.^1^ Recent progress in reducing mortality and health loss in sub-Saharan Africa is attributed to an improvement in access to healthcare interventions against leading causes of ill-health, such as malaria, HIV/AIDS, maternal conditions, and diarrhoeal diseases.^2^ However, the majority of the poor in sub-Saharan Africa still remain without access to healthcare services, hampering greater progress in the healthcare sector.^3^

In sub-Saharan Africa, access to healthcare in both public and private sectors is largely dependent on a patient’s ability to meet OOP expenses at the point of care. Insurance arrangements are virtually non-existent in many health systems. The role of OOP payments in influencing access has been a subject of growing academic and policy interest.^1,4-6^ Since the early 2000s, several African countries have abolished user fees in an effort to reduce financial barriers to access to needed healthcare, especially for the poor.^7-10^ User fees were considered to pose a significant barrier to access to appropriate health services The government of Zambia abolished user fees on outpatient primary healthcare services, firstly in 2006 in rural areas, and extended the policy to urban areas in 2012. The goal of this policy was to increase utilisation and access to health services in both rural and urban primary healthcare facilities.^11,12^

Despite a notable increase in utilisation of public health services following the removal of user fees as reported in studies,^7,13^ a number of questions remain unanswered. For example, some evidence has shown that the impact of user fees on removing barriers to access, on healthcare utilisation, and on illness-related healthcare spending among households, remain unclear. Furthermore, studies have shown that even when user fees have been abolished, individuals and households still incur costs when they visit health facilities.^9,14,15^ When patients turn up at a health facility to seek treatment, they often face uncertain OOP charges. In such a system, the implications for health service utilisation and health expenditure associated with visits to a health provider remain unclear.

This study seeks to contribute to the literature on the relationship between healthcare access and OOP healthcare payments in Zambia where user fees have been abolished. Many empirical studies have analysed the effects of OOP payments, particularly user fees, on healthcare access.^7,6,10^ However, to date, there are only a few studies that have analysed OOP payments and outpatient utilization in a context of free public healthcare in sub-Saharan Africa.^13^ In Zambia, there is no such published study yet. This study analyses OOP payments in Zambia, with a focus on identifying the significant determinants of OOP payments and utilization, following the removal of user fees countrywide in 2012. The main goal of this paper is as follows: (*i*) to examine the factors associated with healthcare choices among the sick people, and (*ii*) to assess the determinants of the magnitude of OOP payments related to a visit to a primary health provider. Our study is based on a nationally representative household-based health expenditure and utilisation survey conducted in 2014.

## Brief Description of the Zambian Healthcare System

Health services in Zambia are provided by three main players, namely the government, the church missionaries (or faith-based facilities) and private-for-profit providers. Public health facilities are the main choice of healthcare for the majority of Zambians. Several national surveys show that over 80% of individuals who sought formal healthcare after falling ill had visited a public health facility.^16-19^ The private healthcare sector is still small but growing.^11,20^ Utilisation of traditional medicine is low in Zambia compared to other countries in sub-Saharan Africa, accounting for less than 1% of total health service provision.^19^

In terms of financing, the Zambian health sector has got multiple sources. The public health system is funded mostly by public funds, local and international donors, and patient fees which still apply in non-primary public health facilities. Although built by Church missionaries, faith-based health facilities depend heavily on the government for their operating budget and occasional financial aid from overseas donors. There is very limited health insurance in Zambia, restricted mostly to employer-based medical insurance schemes.

The public health system is structured as a pyramid with health posts at the bottom as the first point of contact. Health posts are designed to offer basic primary health services such as health promotion (eg, sanitation, nutrition, bed net distribution, immunisation, etc) and basic curative care (eg, treatment of simple malaria, oral rehydration therapy for diarrhoea, etc) at community level. Health posts are usually managed by a public health officer (called environmental health technologist). At the health centre level, a slightly greater scope of services is provided though still basic. The staffing profile of health centres typically includes a clinical officer, a laboratory technician, a pharmacist, nursing staff, midwives, and an environmental health technologist. Most health centres only serve as outpatient facilities. District hospitals provide slightly more advanced curative care and basic surgical services although they are still considered to be part of primary healthcare. In terms of staffing, a district hospital has medical doctors, usually of a general specialty. Above the district hospital level of care are second level hospitals or general hospitals, which are the main referral centre for all district hospitals in each province. At the top of the health system are the third level hospitals, or tertiary level hospitals, which provide clinical services with specialists as well as academic training and research.

## Data and Methods

### Data 

The statistical analyses in this study are based on a cross-sectional dataset from the Zambian Household Health Expenditure and Utilisation Survey (ZHHEUS) conducted in 2014 by the Central Statistical Office. The ZHHEUS sampled a cross-section of households in all 10 provinces of Zambia using a sampling design, which was aimed at achieving national representativeness. The Central Statistical Office, with support from the Ministry of Health, Lusaka, Zambia and the University of Zambia, Lusaka, Zambia conducted the survey, yielding a total of about 12 000 households, including some 59 500 individuals, in all 10 provinces of Zambia. A two-stage stratified cluster sample design was used. In the first stage, standard enumeration areas were selected within each stratum using the probability-proportional-to-estimated-size procedure to select a total sample of 599 clusters or primary sampling units (*psu*) from each of Zambia’s 10 provinces of which 250 were from urban areas and the rest (ie, 349 *psu*’s) from rural areas. A full census (or listing) of all households in each *psu* was conducted prior to sampling of sample households. In the second stage, a fixed proportion of 20 households were selected from each *psu* using a systematic random sampling procedure. Thus, the sample size was powered to be representative at the cluster, provincial and national levels. The survey response rate was 99.4%.

At each sampled household, all members were enumerated for all modules except for the maternal health section, which was restricted to female members aged between 12 and 49 years. The survey included modules on health status (self-rated health status and self-reported illness experience); illness experiences associated healthcare utilisation (visits, admission, type of providers sought, health expenditure); and a quality of care assessment. Specifically, individuals were asked if they had experienced any illness or injury in the 4 weeks preceding the survey, or if they had been admitted to a health facility in the 6 months preceding the survey. OOP health expenditure included charges for consultation, drugs, medical investigations, and other fees incurred at facilities, as well as transportation costs and other costs related to a visit to a health facility.

### Theoretical Approach 

The empirical model applied in this paper is based on the Grossman model of demand for health and healthcare, which describes how individuals make choices regarding healthcare utilization.^21,22^ In the Grossman theoretical framework, utilisation of healthcare is optimally chosen as an attempt to attain or maintain optimal health. When individuals fall sick, they demand healthcare in order to restore their health capital. An important contribution of the Grossman model is in providing a theoretical framework for testing the relationship between characteristics of an individual and his or her health behaviour. Since Grossman, empirical studies have examined the marginal effects of characteristics such as income, age, education, health insurance, health status, distance to a health provider, and so on, on health decisions and healthcare consumption.^23-25^

The Grossman model postulates that apart from expanding an individual’s ability to pay, higher wages lead to a substitution of medical consumption for time or resources invested in health promotion or prevention. In other words, a higher wage induces an individual to dedicate less time to health promotion or prevention and more time to earning a wage.^21,22^ In contexts where healthcare utilisation is dependent on OOP payments, income works through price to relax the consumer’s budget constraint. Hence, income is expected to increase the likelihood of seeking healthcare as well as the magnitude of health spending. In this survey, we used household consumption expenditure which is widely considered to be a more reliable measure of household wealth than self-reported income. It is less sensitive to short-term fluctuations. Consumption expenditure also captures the value of home production, which is important to appropriately measure wealth or economic capacity in many rural settings.^26^

Although Grossman had predicted a negative relationship between education and demand for healthcare on account that education increases an individual’s health prevention ability, through health knowledge, healthy lifestyle, processing health information, and so on, which should imply less need for medical care consumption, empirical studies have shown a positive relationship between education and healthcare utilisation.^27,28^ Empirical studies hypothesise that more years of schooling make individuals choose better healthcare options which include the ability to seek effective medical care following an illness experience.

With regard to age, theory predicts that with increasing age, more healthcare is needed to offset the effect of increasing depreciation of health capital. However, studies have suggested a non-linear relationship as at some point in age, the marginal cost of investing in renewing health exceeds its marginal benefits, at which point this relationship becomes negative.^29,30^ Also, the literature shows the demand for healthcare to be higher among children under the age of five years and among the elderly.^31^

Empirical extensions of Grossman’s work have included other factors such as gender and region of residence. In this paper, the set of explanatory includes gender, age, household per capita consumption expenditure, highest level of education attained by the head of the household, employment status of the household head, residential location of the household, type of illness reported by an individual and the type of healthcare provider visited. Household consumption expenditure was used as a proxy for household income or wealth. Table 1 provides the full list of variables used and their definitions.

**Table 1:** Definitions of Variables

**Variable Name**	** Variable Description**
Dependent variables	
Care options	Healthcare option chosen by those reporting illness:
	1. Formal healthcare
	2. Self-medication (including traditional)
	3. Did nothing
OOP health expenditure	Health expenditure during a visit including expenditure on expenses for consultation, drugs, medical investigations, registration and transportation. Unit of measurement is ZMK
Independent variables	
Monthly household per capita expenditure	Household per capita expenditure per month in ZMK
Employment status	Type of employment of household head (Salaried employee = 1, 0 = Self-employed, unpaid household worker, intern, student, and other)
Level of Education	Education of household head, highest level of schooling completed:
	1. No formal education
	2. Primary level (1 to 7 years of schooling)
	3. Secondary level (8 to 12 years of schooling)
	4. Tertiary level (college, university, etc)
Illness symptoms	Type of illness symptoms self-reported by patient
Malaria/fever	Patient reported to be suffering from malaria or fever
Respiratory illnesses	Patient reported to be suffering from respiratory diseases
Headache	Patient reported to be suffering from a headache
Diarrhoea	Patient reported to be suffering from diarrhoea
Other illnesses	Patient reported to be suffering from other illnesses apart from malaria/fever, diarrhoea, headache, or respiratory infections
Provider type	Type of healthcare provider chosen:
	1. Public hospital
	2. Public health centre
	3. Public health post
	4. Mission health facility
	5. Private health facility
	6. Other health facility types
Distance from home to facility	Distance covered to and from health facility in kilometres
Region of residence	Place of residence for a household (Rural = 1; Urban = 0)
Age in years	Age of person in years
Gender	Gender of person (Male = 1; Female = 0)

Abbreviations: OOP, out-of-pocket; ZMK, Zambian Kwacha.

### Analytical Models and Estimation 

Empirically, our approach leads us to estimate two models. First, we specify a multinomial logistic regression model of an individual’s decision regarding healthcare utilisation. In the second part of the analysis, we analyse determinants of the magnitude of OOP healthcare expenditure conditional on visiting a health provider using a two-part estimation procedure. The logistic regression to estimate the probability of an individual incurring a positive expenditure and the generalised linear model to analyse the determinants of the magnitude of OOP healthcare expenditure.

### Determinants of Healthcare Utilisation

Given that the response variable is at three mutually exclusive levels (sought formal care, performed self-medication, or did nothing), and our intention to model effects (on the response variable) that operate at the community level, we fit a multilevel multinomial logistic regression model. In a multinomial logistic model, the probability of an individual *i* living in primary sampling unit *j*, choosing care option *p* is given by $\pi_{ij}^{p}$ *= Pr (care option* *= p)*. Thus, *p* *= *1,..,*q* (*q* *= *3). One of the response categories is taken as the reference category. In this case, the “did nothing” was the “reference” response category. We estimated a simultaneous set of *q*-1 logistic regressions for the other two “care option” categories, contrasting each category with the reference category. Thus, a separate intercept and slope parameter was estimated for each of the categories, as indicated by the *p* superscripts.

The multilevel multinomial logistic regression model is specified using the following logit link:

$$\log it\left(\frac{\pi_{ij}^{p}}{1-\pi_{ij}^{p}}\right)\beta^{p}X_{ij}+u_{j}^{p}+\varepsilon_{ij}^{p},p=1,...q-1$$

Probability,π_ij_^p^, is a function of a vector of covariates denoted by *X*, and the specified community level (i.e. *psu*) random effects (u_j_^p^). The term represents random variation in the likelihood of doing nothing relative to formal care, or doing nothing compared to self-medication, at *psu* level. The parameter *β*^(p)^ represents the fixed part of the model which is interpreted as the change in the odds of being in category *p* relative to the “reference” category associated with a 1‐unit increase in the explanatory variable denoted by *X,* if *X* is continuous. In the case of discrete explanatory variables, *β*^(p)^ represents the change in the odds associated with being in one category (eg, living in a rural area) relative to being in the reference category (being in an urban area). The model assumes that u_j_ ~ N(0,σ_u_^2^). Further, the residual error term denoted $\varepsilon^{\frac{p}{ij}}$ is random error at individual level which is assumed to have a logistic distribution with mean zero and variance $\frac{\pi^{2}}{3}$ .

In this hierarchical structure, we take account of variations in choice of care option that operate not just at the individual or household levels but also at the community level. Community level or neighbourhood in this case is defined by the survey clusters called primary sampling unit (*psu*). It is plausible that observed variations in healthcare choices might be partly explained by community level influences on health-related behaviour.^32-34^ In this approach, unobserved variations in healthcare choices are captured as random effects operating at the community level through the parameters *u*_j_.^32^ The model was estimated using the maximum likelihood method in the generalised linear latent mixed model (gllamm) framework. The gllamm procedure provides an estimation algorithm that is more robust than either ordinary least squares (OLS) or traditional maximum likelihood estimators.^32^

### Determinants of Outpatient Out-of-Pocket Health Expenditure

In this part of our analysis, we model the determinants of the magnitude of outpatient OOP healthcare expenditure. In the survey, only individuals who reported a visit to a health provider were asked about expenditure incurred during a visit. Those who did self-medication were not asked to state how much they may have spent. In estimating the health expenditure model, we considered a number of methodological challenges commonly reported in the literature.^35,36^ Specifically, the distribution of OOP expenditure shows a high density at zero and a right-skewed continuous distribution of positive amounts. These findings are because, as stated earlier, user fees at all public and mission primary level healthcare facilities have been abolished for primary health services. For example, if an individual did not incur any transportation (ie, if they may have walked to the facility) or other health visit related expenses, their total health expenditure for the visit would be zero. Thus, the zeros in the data are real zeros, and not due to censoring (censoring typically referring to reporting a zero OOP expenditure simply because an individual did not fall sick or did not seek care) but akin to a semi-continuous dependent variable problem.^37^ In such a case, applying the standard OLS estimation procedure would result in biased and inefficient estimates.^35^

Based on guidance from the literature,^35,36,38,39^ we applied a two-part estimation procedure. The first part of this model constructs a logistic regression to estimate the probability of an individual incurring a positive expenditure among, which is expressed as follows:

$$\log\left[\frac{\mathrm{Pr}(OOP>0\backslash x)}{1-\mathrm{Pr}(OOP_{i}>0\backslash x)}\right]=x\beta +\varepsilon_{i}$$

where *OOP*_i_ is the level of OOP expenditure on an outpatient visit by individual *i*, *x* is a vector of covariates (as defined earlier, plus distance travelled to a facility, in kilometres), *β* denotes coefficients of the corresponding estimates, *ε*_i_ is the stochastic error term. The second part of the model predicts the magnitude of OOP expenditure for a visit, conditional on expenditure being positive, using the set of explanatory variables identified above. In this part of the model, we estimate the expected OOP expenditure given the same set of explanatory variables as defined earlier (with the inclusion of distance travelled), denoted by *x*. Thus, *E*(*OOP\x*) = *xβ*.

In estimating the second part of the model, we considered the generalised linear model (*glm*) and OLS estimators, taking into account the trade-offs in bias and efficiency each estimator brings. Given a kurtosis coefficient on the log-scale residuals of the *glm* estimator of 3.18 and the overall superiority over OLS, we chose the *glm* estimator.^40^ Specification of the *glm* framework requires the analyst to choose a link function that models how the dependent variable is connected to the explanatory variables, and a distribution function that models the relationship between the mean and variance of the expectation of the dependent variable. We tested alternative link-distribution combinations appropriate for the type of data at hand. The best fitting model was the *glm* with a gamma distribution and a log link function. The gamma-log *glm* yielded the lowest value of the Akaike information criterion (AIC) (2.9) and the deviance statistics (7483.7) compared to other *glm* specifications. It is also theoretically a more appropriate fit for our data.^41^ To improve on model efficiency, robust standard errors were clustered at the primary sampling unit (*psu*) level. All computations were done using the *tpm* routine in Stata 13.

## Results

### Descriptive Statistics of the Sample

Table 2 presents the descriptive statistics of the sample. Following an illness or injury, about 60% of survey respondents chose to consult a healthcare provider. A further 30% opted for self-medication, while 10% did not seek for healthcare. Public health facilities remained the main choice of provider for reported visits to health providers (13% at public hospitals, 57% at public health centres, 20% at health posts). Mission facilities accounted for about 6% of visits to health facilities. Only 3.2% reported having visited private health facilities, while visits to traditional practitioners and other sources of care each accounted for 2% of visitors.

**Table 2:** Sample Descriptive Statistics

**Variable Name**	**Statistic**
Region of residence	
% Living in rural areas	60.2
Head of household in formal employment	
% In formal employment	21.3
Monthly household per capita consumption expenditure, n = 11 595	
Mean (SD)	214.7 (498.6)
Median	78.0
Level of education of head of household (%), n = 11 595	
No formal schooling	21.3
Primary (1-7 years of schooling)	47.7
Secondary (8-12 years of schooling)	26.5
Tertiary (post-secondary school training)	4.5
Age in years, n = 59 514	
Mean (SD)	21.7 (17.9)
Median	17.0
Gender, n = 59 514	
% Female	51.1
Prevalence of illness, n = 59 514	
% Reporting an Illness in the four weeks preceding survey	22.1
Care options (%), n = 13 149	
Formal healthcare	59.4
Self-medication	29.9
Do nothing	10.7
Type of illness (%), n = 13 149	
Malaria/fever	49.0
Respiratory	4.2
Headache	13.2
Diarrhoea	5.7
Other	27.9
Health provider type visited (%), n = 7923	
Tertiary or secondary level hospital	3.7
District hospital	9.1
Public health centre	57.1
Public health post	19.0
Mission health facility	5.9
Private health facility	3.2
Distance to and from facility in Kilometres, n = 7923	
Mean (SD)	10.5 (26.0)
Median	1.5

The most common cause of illness reported was malaria and/or fever illness at 49%. Other leading causes of illness were headache (13.6%), diarrhoea (5.7%), and diseases of the respiratory system (including pneumonia) (4.2%). A larger proportion (60.2%) of the sample resides in rural areas. The average distance travelled, to and from, the facility visited during the last illness episode was 5.2 km, with a maximum of 200 km. The minimum shows zero but could have been measurement errors reflecting that the distance may have actually been less than a kilometre. The median (mean) age of the sample was 17 (21.7) years.

Average per capita monthly household consumption expenditure was Kwacha 214.70 (equivalent to about US$35). In terms of employment, 21.7% of the respondents were salaried employees, the rest were self-employed, unpaid household worker, students or seeking work. About half of heads of households have attained only up to primary education (ie, 1-7 years of schooling). One in five households are headed by individuals who have no formal education while only 4.3% of heads of households have at least post-secondary education (typically college or university degrees). These statistics are consistent with the statistics reported in the Zambia Demographic and Health Survey (2013).^19^

In terms of OOP expenditure associated with a visit to a healthcare provider, the estimated mean OOP expenditure was K14.90 (approximately US$2.50) for the entire sample of patients who visited a provider, ie, including those who reported spending nothing (Table 3). The mean of those who reported positive amounts was significantly higher at K79.50 (US$13.03). The difference between these two mean expenditures appears significant because about 80% of individuals who visited a public health provider did not incur any OOP expenses due to the policy of no user fees at primary healthcare level. The median expenditure was K0.00 for entire sample of patients who visited a health provider because as indicated earlier 80% did not incur any expenses. For those who reported positive expenditure the median was K9.00 (US$1.46). Furthermore, Figure shows that the burden of health expenditure is much higher among the poorest households. For example, OOP expenditure represented 41% of total expenditure among the poorest quintile while the mean OOP expenditure as a proportion of total household consumption expenditure was 3.4% in the richest quintile.

**Table 3:** Description of OOP Health Expenditure

**Variable**		**Statistic**
OOP health expenditure including those with Zeros	Mean	14.9
	Median	0.0
	Min	0.0
	Max	10.278
	95% CI	(11.912-17.821)
OOP health expenditure including only those with positive expenditure	Mean	74.9
	Median	8.0
	Min	0.5
	Max	10.278
	95% CI	(60.252-89.600)

Abbreviation: OOP, out-of-pocket.

**Figure:**
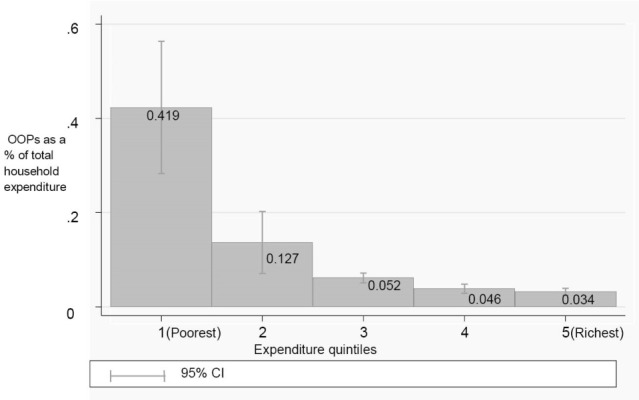
Out-of-Pocket (OOP) Health Payments as a % of Total Household Expenditure, by Expenditure Quintile.

### Determinants of Healthcare Utilisation

In Table 4, results of the multilevel multinomial logistic regression of the factors associated with decision whether to seek care, self-medicate or do nothing are presented. In the multinomial logit, the category ‘sought formal healthcare’ (C1) and ‘self-medication’ (C2) options are compared with the reference category ‘did nothing’ (C3). Coefficients have been transformed into odds ratios (ORs). A number of socio-economic and demographic factors are shown to have significant effects on the decision whether to seek care or not. Household consumption expenditure is significantly associated with increased odds (or likelihood) of seeking formal care following an illness relative to doing nothing (OR = 1.119; 95% CI: 1.060-1.183). A 10% increase in per capita consumption expenditure is associated with an increase by a factor of 0.01 units in the odds of seeking formal care relative to doing nothing. Further, households with higher consumption expenditure have increased odds of choosing self-medication relative to doing nothing (OR = 1.149; 95% CI: 1.083-1.218).

**Table 4:** Multinomial Logit Model of an Individual’s Choice of Care Option

**Care options**	**OR**	**95% CI**
Sought care (C1)		
Region of residence (rural = 1, urban = 0)	0.706***	0.569-0.876
Age in years	0.989***	0.986-0.991
Gender (male = 1, female = 0)	0.924	0.818-1.043
Log of household per capita expenditure	1.119***	1.060-1.183
No formal education (reference category)	-	-
Primary level	1.417***	1.176-1.707
Secondary level	1.536***	1.242-1.900
Tertiary level	1.547**	1.105-2.164
Malaria or fever (reference category)	-	-
Respiratory illnesses	1.011	0.742-1.378
Diarrhoea	0.777*	0.595-1.015
Headache	0.570***	0.478-0.680
Other illnesses	1.276	0.761-2.140
Employment status	0.909	0.737-1.121
Constant	6.279***	4.378-9.005
Self-medication (C2)		
Region of residence (rural = 1, urban = 0 )	0.583***	0.467-0.729
Age in years	0.990***	0.987-0.993
Gender (male = 1, female = 0)	1.125**	0.988-1.280
Log of household per capita expenditure	1.149***	1.083-1.218
No formal education (reference category)	-	-
Primary level	1.341***	1.097-1.639
Secondary level	1.358***	1.081-1.705
Tertiary level	1.542**	1.083-2.196
Malaria or fever (reference category)	-	-
Respiratory illnesses	1.220	0.878-1.695
Diarrhoea	1.308*	0.992-1.724
Headache	1.440***	1.206-1.720
Other illnesses	1.184	0.679-2.064
Employment status	0.726***	0.582-0.906
Constant	2.715***	1.853-3.978
Did nothing	(Base outcome)	
***level 2 (psu1) Variance (1): 0.499 (.068)		

Abbreviation: OR, odds ratio. *** *P* < .01 (significant at 1%), ** *P* < .05 (significant at 5%); * *P* < .1 (significant at 10%). Number of level 1 units = 12 858; Number of level 2 units = 596; Condition Number = 851.3.

The results show that the odds of seeking formal care (relative to doing nothing) increase with the level of education of the head of the household. The relative odds of seeking formal care rather than doing nothing is 42% higher for individuals living in households in which the head has primary education than for individuals from households where the head has no formal schooling. The odds of seeking formal care are even greater for individuals living in households in which the head has secondary (OR = 1.536; 95% CI: 1.242-1.900) or tertiary (OR = 1.547; 95% CI: 1.105-2.164) levels of education. Furthermore, the odds of self-medicating rather than doing nothing increase by between 34% and 54% if an individual comes from a household headed by someone with some level of education (primary, secondary, or tertiary) compared with a household where the head has no formal schooling.

Formality of employment of the household is not shown to influence the odds of seeking formal care relative to doing nothing. However, having a head of household in formal employment is associated with a reduced likelihood of using self-medication compared to doing nothing (OR = 0.726; 95% CI: 0.582-0.906). Having either headache or diarrhoea or ‘other illness’ was associated with reduced odds of seeking formal healthcare (relative to doing nothing), compared with reporting malaria or fever. For example, diarrhoea illness (self-reported) was associated with a reduction by a factor of 0.78 in the odds of seeking formal care. However, those who reported having diarrhoea had 31% higher odds of self-medicating (relative to doing nothing) than those who reported symptoms of malaria or fever. Co-morbidities were captured under other illness types, although less than 1% reported having more than one symptom. Rural residence is associated with a significantly lower likelihood of seeking formal care instead of doing nothing (OR = 0.706; 95% CI: 0.569-0.876). The odds for resorting to self-medication (relative to doing nothing) were even lower for those living in a rural area compared to those living in urban areas (OR = 0.583; 95% CI: 0.467-0.729).

The association between age and the likelihood of seeking formal care compared to doing nothing is negative and significant though small in magnitude. Younger patients are also more likely to self-medicate than do nothing. However, the association between gender of the patient and the care options is largely insignificant, although the odds for self-medicating relative to doing nothing are higher among men than among women. This result is statistically significant at the 10% level. Finally, the variance is decomposed into a between-cluster variance (1.76) and the residual (within-cluster) variance (1.41). The estimated Intra-cluster correlation coefficient (ICC), calculated as ICC = 0.499/(0.499 + 3.293), indicates that 13% of the total variation in healthcare choice (ie, decision to seek care, self-medicate or do nothing) is explained by cluster level factors which are measured by the cluster (*psu*) random effects.

### Health Expenditure Analysis

Results of the two-part model used to estimate determinants of health expenditure during a visit are shown in Table 5. First, we analyse the results from the first part of the table which presents the logistic regression part of the results. A number of factors are shown to be related with the odds or likelihood of incurring OOP payments. Per capita household consumption expenditure was significantly and positively associated with an increased likelihood of incurring OOP payments during a visit to a health provider. Rural residence was negatively related with the odds of incurring OOP payments. Visiting a public health centre, a public hospital or a private facility was strongly associated with increased odds of incurring OOP payments compared to visiting a health post. In addition, the farther away the individual from the facility, the higher the odds of incurring OOP payments. Increasing age of the patient was also shown to be related with an increased likelihood of incurring OOP payments. Generally, the level of education of the head of the household, type of illness and gender of the individual were not shown to influence the odds of incurring OOP payments.

**Table 5:** Two-Part Model Estimation of OOP Health Payments

Number of observations = 7739, Clusters = 591
Wald chi2 (18) = 328.7, Prob > chi2 = 0.000		
Log pseudo likelihood = -4544.3, Pseudo R2 = 0.075
**Variables**	**OR**	**95% CI**
Distance to health facility	1.010**	1.002 1.018
Age in years	1.007***	1.005 1.010
Gender	1.026	0.933 1.130
Region of residence	0.805*	0.651 0.997
Log of household per capita expenditure	1.387***	1.296 1.484
No formal education (reference category)	-	-
Primary level	1.119	0.882 1.419
Secondary level	1.033	0.805 1.325
Tertiary level	1.168	0.830 1.644
Malaria or fever (reference category)	-	-
Respiratory illnesses	1.168	0.884 1.543
Diarrhoea	1.008	0.786 1.292
Headache	0.932	0.766 1.133
Other illnesses	1.254***	1.099 1.431
Public health post (reference category)	-	-
Tertiary/secondary hospital	1.547***	1.232 1.942
District hospital	2.175***	1.609 2.941
Public health centre	1.262***	0.862 1.848
Mission health facility	0.121	0.097 0.140
Private/other health facility	3.747***	3.133 4.001
Employment status	0.929	0.892 1.025
Constant	0.070***	0.068 0.110
**Generalised linear model estimation**		
Number of observations = 2559		
Clusters = 526		
Deviance = 7263.7		
Pearson = 43 782.6		
Bayesian information Criterion = -12 514.5		
AIC = 9.81		
Log pseudo likelihood = -12 532.3		
**Variable**	**Coefficient**	**SE adjusted for clustering on psu**
Distance to health facility	0.013***	0.003
Age in years	0.025***	0.003
Gender	-0.358*	0.154
Region of residence	0.317*	0.177
Log of household per capita expenditure	0.147**	0.069
No formal education (reference category)	-	-
Primary level	0.429**	0.117
Secondary level	0.680***	0.223
Tertiary level	0.266	0.268
Malaria of fever(reference category)	-	-
Respiratory illnesses	-0.053	0.249
Diarrhoea	0.018	0.219
Headache	-0.543**	0.218
Other illnesses	0.273	0.172
Public health post (reference category)	-	-
Tertiary/secondary hospital	1.650***	0.323
District hospital	0.479**	0.212
Public health centre	0.245	0.218
Mission health facility	0.542	0.285
Private/other health facility	1.414***	0.275
Employment status	0.568*	0.292
Constant	1.352***	0.428

Abbreviations: OR, Odds ratio; OOP, out-of-pocket; AIC, Akaike information criterion; SE, standard error. *** *P* < .01, ** *P* < .05, * *P* < .10.

The second-part of Table 5 shows results of the *glm* with log link. Household consumption expenditure is positively and significantly related with the magnitude of OOP payments. The coefficient on logarithm of per capita consumption expenditure indicates that a 10% increase in per capita consumption expenditure is associated with an increase in OOP payments by a factor of 0.014 Kwacha. The associated elasticity shows that a 10% increase in total consumption expenditure is associated with a 0.2% increase in OOP payments. Distance is also shown to be a significant predictor of the level of OOP health spending, with every kilometre travelled being associated with a K0.51 increase in OOP payments. Having a head of household with primary or secondary education was associated with a higher level of OOP payments. The type of illness reported did not generally affect the magnitude of healthcare spending. However, the level of OOP payments increased with the level of care. For example, visiting a public health centre or a district hospital was associated with a significantly higher OOP payments compared with visiting a health post. District hospital visits were associated with a significantly higher OOP payments compared to health posts. Further, those who sought care at a tertiary or secondary public hospital spent significantly more than those who visited a health post. Even though OOP payments at faith-based facilities were higher than at a health post, the differences were not statistically significant. In addition, a visit to a private healthcare provider was associated with a significantly higher OOP payments than a visit to a health post. Finally, having a head of the household in formal employment is associated with a higher level of OOP payments.

## Discussion

This study investigated the key factors that determine utilisation of outpatient health services and the associated OOP spending in Zambia. According to the findings from the multinomial logistic regression, several socio-economic variables are shown to influence the likelihood of seeking formal care rather than doing nothing or self-medication. For example, the likelihood of consulting a formal healthcare provider relative to doing nothing about an illness was generally greater among individuals from wealthier households. This finding confirms that households with very limited financial means or none at all, are unlikely to seek care because of the perceived or real financial commitment that comes with formal healthcare utilisation. The importance of household economic capacity in influencing the decision to seek formal healthcare utilisation is consistent with what an earlier study in Zambia^42^ and other studies elsewhere have found.^7,43^

Similarly, the level of education of the head of the household was positively associated with a greater demand for formal healthcare relative to doing nothing about an illness. As hypothesised in other empirical healthcare demand studies, education increases an individual’s ability to acquire and utilise health information.^27,44^ Further, the negative association between rural residence and the likelihood of seeking formal care, after controlling for household expenditure, education of the head of the household and other factors, reflects the generally poorer access to health facilities in rural areas. Overall, the importance of these socio-economic factors in influencing the decision to seek care suggests the existence of significant socio-economic inequality in healthcare access.

This study further demonstrates that the type of perceived illness is a significant factor in the individual’s decision to seek formal care or not. Suffering from malaria or fever illnesses was associated with a greater likelihood of seeking formal care compared with diarrhoea and headache. Perceptions about availability of effective treatment either at home or at a formal healthcare facility may drive the decision whether to seek formal care or not. Literature shows that in Africa, the decision to seek formal care or self-medicate can be dependent on the type of perceived illness.^45,46^ It is important to bear in mind the survey did not ask about severity of the illness. Nonetheless, we would recommend that this issue be subject to further investigation in future studies. In the analysis of the determinants of the level of OOP payments, the study has shown that OOP payments is significantly associated with household economic well-being, education of the head of household, distance to health facility, region of residence, employment status of the head of the household, age and gender of the patient, and the type of facility visited. Several aspects of the Zambian health system may help explain the above findings in terms of OOP health expenditure. With regard to household economic well-being, it is quite common that when public health facilities run out of drugs, patients often have to buy drugs at pharmaceutical retail outlets. Patients are also often asked to have their medical investigations conducted at a private facility after having initially visited a public health facility. In such cases, ability to pay is likely to influence the magnitude of OOP payments. The poor might not afford the cost of drugs at retail outlets or medical investigations in private facilities, and are more likely to skimp on quality or quantity of services.^9,10^

There are also other ways in which OOP payments for healthcare might be positively associated with household economic resources. For example, individuals with less resources may have used a mode of transportation that does not cost money (eg, walking or using a bicycle), while the non-poor might have actually spent more on travel-related expenses in search of facilities with better quality care (shorter queues, cleaner surroundings, more courteous staff, etc). Another possible factor is the possibility of informal or unofficial user charges. Although this may not be definitive evidence of informal user fees, data shows that some individuals had paid fees at health posts and health centres, both of which fall under the classification as primary healthcare facilities. Possibilities of unofficial user charges in the context of free healthcare have been cited in Africa.^10^ Further research is required to establish whether implementation of user fee removal policy follows official guidelines.

A possible explanation for the positive association between education and the magnitude of OOP payments could be that better-educated individuals may have a tendency to choose better-quality, more costly healthcare options.^44,46^ Similarly, in a setting such as Zambia where there is no widespread health insurance, formal sector employment status provides a more effective means to afford healthcare payments. In terms of the influence of provider type, a visit to hospitals is associated with higher OOP payments largely because of more advanced treatment and longer distances (on average a patient resides nearer a health post or health centre than a hospital).

Consistent with other studies, this study shows that people who reside in remote and rural areas generally live further from health facilities which increase their OOP payments through travel cost.^14,47^ A significant component of OOP payments for people residing in rural areas and remote settings is the cost of travel to access health services. Clearly, OOP payments increases with increasing remoteness. In fact, it has been demonstrated that even in the context of free health services at the point of care, geographical barriers to (and invariably the cost of) access remain an issue.^46^ The finding that private facilities and higher level public facilities are associated with higher OOP payments might reflect either better quality or higher user fees or both, at these facilities.

Taken together, findings from this study provide new evidence on the existence of significant financial burden of OOP payments for patients from the poorest households in Zambia. Clearly, the policy goal of reducing financial burden of access to care seems to have been only partially achieved, as our findings show that among those who incurred an expense (20% of those who reported an illness), OOP payments as a percentage of total expenditure averaged 40% in the lowest quintile of richness. Furthermore, this study suggests that the benefits of the policy of free primary healthcare in the public sector disproportionately accrue to the population that resides in more urbanised areas with generally better physical access to better-resourced health facilities. Studies elsewhere in Africa have documented the limited impact of user fee removal policy in increasing utilisation or reducing financial barriers to access on account of some of the factors described above.^9,10^ With the recent policy impetus towards universal coverage across health systems in Africa, the question of effective policy responses to reducing inequalities in access to quality health services has become increasingly important.

## Conclusion

This study provides new evidence from a national survey of 2014 (N = 59 500 respondents) on the determinants of utilisation of outpatient health services in Zambia. A total of 80% of patients who visited public primary healthcare facilities on an outpatient basis reported not having incurred any medical, travel or any other healthcare-related OOP expenses. This finding confirms that implementation of user fee removal policy has been routinized in the Zambian public health system.

However, the study also demonstrates that utilisation of formal healthcare is strongly related with a number of socio-economic factors. Additionally, factors such as distance, provider type and the patient’s socio-economic status represent the major drivers of the magnitude of OOP payments. The study shows that the burden of OOP expenditure remains considerably higher among the poorest households. For example, the cost of long distances to facilities hurts the poor more because the poor are more likely to live farther away from health facilities.

The findings from this study raise several implications for policy especially regarding equity of access and universal health coverage for the Zambian healthcare system. First, the study demonstrates a need for policy attention to other factors, notably long distances to facilities that constrain access to healthcare. Policy attention should focus on increasing physical access. At present, the poorest who reside in remote parts of the country are not benefiting from free primary health services in the public sector. Second, efforts to improve quality of care through provision of adequate drugs and reducing waiting time would be crucial to achieving greater access. Third, the government should investigate implementation of the policy of free primary health services to ensure that the policy is being implemented as intended. Given that the data shows that some patients reported OOP payments for registration, consultation, drugs or diagnostic services, and so on, at public primary healthcare facilities, which should be free, is a worrying phenomenon. In addition, understanding the factors that influence the high prevalence of self-medication would be important for policy given the potential risks associated with poorly regulated pharmaceutical markets in developing countries. Finally, an analysis such as a benefit incidence analysis would be useful to determine whether the poor have benefited equitably from free primary healthcare policy.

## Ethical issues

Ethical exemption for this study was granted by the Central Statistical Office under the provisions of the Census and Statistics Act Number 127 of the laws of Zambia, since the study did not involve collection of human samples. No identifying information of individuals or health institutions were collected in the survey. Furthermore, in observance of ethical requirements, only participants aged at least 15 years were interviewed after giving written (signature or thumb print) informed consent.

## Competing interests

Authors declare that they have no competing interests.

## Authors’ contributions

FM conceived the study; both authors contributed to the design of the study, data analysis, and drafting of the manuscript. Both authors have reviewed and approved the final version of the manuscript and revisions of the manuscript.

## Key messages

**Implications for policy makers**

- Given that the majority of the sick people in Zambia utilise public health services and 80% of whom did not incur any out-of-pocket (OOP) payments suggests that removal of user fees has alleviated the cost of access to healthcare among sections of the population.
- However, this study demonstrates that financial burden of OOP payments targets more the poorest sections of the population. This is because those who live in rural and remote areas still face significant financial and non-financial barriers to access to public primary health even though the services are free. For example, OOP payment in form of travel costs, medical expenses for drugs or investigations not available at public facilities, represent remaining challenges for reducing financial barriers to access.
- Health policy attention should focus on reducing distance to healthcare facilities because distance constitutes a major cost of seeking care.
- To be more effective, the policy of user fee removal must be supported by availability of drugs, basic diagnostic services and human resources.
- An investigation into the nature of medical expenses paid at public facilities is needed to rule out the possibility of informal payments or illegal charges to patients.

**Implications for public**

The study provides new evidence on the costs associated with healthcare utilisation in the Zambian context. The results also demonstrate the remaining challenges to access especially among the poor people, who could be used to lobby for more investments in public healthcare infrastructure. The study also informs the public that public sector remains the largest provider of health services in the country.
